# Editorial: Epigenetic regulation in fibrosis

**DOI:** 10.3389/fmolb.2025.1718307

**Published:** 2025-10-23

**Authors:** Qingqing Wei, Xiaoru Hu, Yan Sanders

**Affiliations:** ^1^ Department of Cellular Biology and Anatomy, Medical College of Georgia, Augusta University, Augusta, GA, United States; ^2^ Department of Rheumatology and Immunology, Xiangya Hospital, Central South University, Changsha, Hunan, China; ^3^ Provincial Clinical Research Center for Rheumatic and Immunologic Diseases, Xiangya Hospital, Central South University, Changsha, Hunan, China; ^4^ National Clinical Research Center for Geriatric Disorders, Xiangya Hospital, Central South University, Changsha, Hunan, China; ^5^ Department of Biomedical and Translational Sciences, Eastern Virginia Medical School, Macon & Joan Brock Virginia Health Sciences at Old Dominion University, Norfolk, VA, United States

**Keywords:** epigenetic regulation, fibrosis, DNA methylation, histone modification, RNA modification, non-coding RNA, biomarker, therapeutic targets

Fibrosis is one of the pathological conditions underlying the progression of numerous chronic diseases, and it contributes substantially to morbidity and mortality worldwide. Increasing amounts of evidence indicate that epigenetic mechanisms—including DNA methylation, histone modifications, RNA modifications, and non-coding RNAs—play pivotal roles in epithelial cell injury and maladaptive tissue repair, inflammatory cell and fibroblast activation, and extracellular matrix remodeling and accumulation. Compared to genetic alterations, epigenetic changes are relatively reversible, positioning them as promising therapeutic targets. The articles collected in this Research Topic highlight recent advances in understanding how epigenetic regulation shapes fibrotic processes across multiple organ systems and provides new opportunities for biomarker discovery and therapeutic intervention.

Pérez et al. reviewed the role of epigenetic modifications in cardiac fibrosis. They described how DNA methylation, RNA modification, histone modifications, and non-coding RNAs governed cardiac fibroblast activation and extracellular matrix deposition, with a detailed discussion of key epigenetic modification enzymes. Importantly, they highlighted emerging pharmacological strategies that targeted critical epigenetic regulators as potential therapies for cardiac remodeling, underscoring the translational promise of epigenetic regulation in cardiovascular disease.

Focusing on pulmonary fibrosis, Huang et al. provided a comprehensive overview of recent mechanistic insights. They outlined how DNA and RNA methylation, histone modifications, and diverse non-coding RNAs regulate pulmonary fibrosis through key processes, including fibroblast activation, epithelial–mesenchymal transition, and immune cell interactions. Their analysis illustrated how different layers of epigenetic mechanisms reinforced profibrotic signaling and revealed potential therapeutic targets for prospective precision medicines in lung diseases.

Chen et al. contributed original research linking epigenetic regulation to chronic kidney disease (CKD). By integrating meta-analysis of clinical cohorts, experimental validation in obstructive nephropathy models, and single-cell transcriptomics, they demonstrated a marked reduction of brain-derived neurotrophic factor (BDNF) in injured kidney proximal tubular cells, macrophages, and podocytes in CKD. Mechanistically, they identified the associated increase of lncRNA Bdnf-as, an epigenetic repressor of BDNF. This study highlighted the value of integrating multi-level approaches to elucidate the role of non-coding RNAs in organ-specific fibrotic progression.

Extending the scope, Niu et al. used transcriptomic and bioinformatic analyses and identified novel biomarkers in age-related macular degeneration (AMD), a condition characterized by oxidative stress, tissue remodeling, and eventual subretinal fibrosis leading to fibrovascular scarring. They highlighted SLFN11 and GRIN1 as candidate biomarkers enriched in immune and stress-response pathways, validated their expression in patient samples, and proposed potential regulatory networks involving transcription factors, microRNAs, and histone deacetylase inhibitors. This work emphasized the shared contribution of epigenetic dysregulation across degenerative pathologies and demonstrated the utility of computational approaches to uncover novel therapeutic targets.

Altogether, these contributions revealed how diverse epigenetic modifications converge on common fibrogenic mechanisms across organs. They also represented methodological breadth—ranging from reviews of the most current research advances to experimental validation and integrative bioinformatics—driving the field forward. Notably, the application of genome-wide bioinformatic analysis, including single-cell and spatial epigenomic technologies, is refining our understanding of cell–type–specific and unbiased changes in fibrotic tissues. Integrating multidisciplinary approaches with experimental validation may identify context-dependent therapeutic targets or biomarkers and support the design of interventions to selectively disrupt pathological fibrotic progression with minimum side effects. The studies in this collection have together enhanced our understanding of fibrotic diseases by advancing both mechanistic insight and translational potential.

